# Secretion of poly-γ-glutamic acid by *Bacillus atrophaeus* NX-12 enhanced its root colonization and biocontrol activity

**DOI:** 10.3389/fmicb.2022.972393

**Published:** 2022-07-29

**Authors:** Jian Xue, Tong Tong, Rui Wang, Yibin Qiu, Yian Gu, Liang Sun, Hong Xu, Peng Lei

**Affiliations:** State Key Laboratory of Materials-Oriented Chemical Engineering, College of Food Science and Light Industry, Nanjing Tech University, Nanjing, China

**Keywords:** *Bacillus atrophaeus*, antifungal, biocontrol, poly-γ-glutamic acid, rhizosphere colonization

## Abstract

*Bacilli* are used as biocontrol agents (BCAs) against phytopathogens and most of them can produce poly-γ-glutamic acid (γ-PGA) as one of the major extracellular polymeric substances (EPSs). However, the role of γ-PGA in plant biocontrol is still unclear. In this study, *Bacillus atrophaeus* NX-12 (γ-PGA yield: 16.8 g/l) was screened, which formed a strong biofilm and has been proved to be a promising BCA against Cucumber *Fusarium* wilt. Then, the γ-PGA synthesis gene cluster *pgsBCA* was knocked out by CRISPR-Cas9n. Interestingly, the antifungal ability of γ-PGA synthetase-deficient strain NX-12Δ*pgs* (γ-PGA yield: 1.65 g/l) was improved *in vitro*, while the biocontrol ability of NX-12Δ*pgs* was greatly diminished *in situ*. Data proved that γ-PGA produced by NX-12 contributes to the biofilm formation and rhizosphere colonization, which effectively improved biocontrol capability. Taken together, these findings prove that the mechanism of γ-PGA promotes the colonization of NX-12 and thus assists in controlling plant diseases, which highlight the key role of γ-PGA produced by BCA in biocontrol.

## Introduction

*Fusarium* wilt is a very serious disease and it is caused by *Fusarium oxysporum* (Dr et al., 2003; Dean et al., 2012). This disease has become one of the main factors restricting cucumber production worldwide as the fungus is present in all types of soil worldwide (Cao et al., 2011). Because of the lack of effective chemical control methods, biological control has become a potential alternative to chemical pesticides and other conventional control methods (Compant et al., 2005; Raza et al., 2016). Biocontrol agents (BCAs) can colonize plant rhizosphere or tissues and therefore resist plant root pathogens through direct action (including the secretion of antibiotics and production of various hydrolases) or indirect action (including nutritional or space competition with pathogenic microorganisms and induction of systemic resistance; Backer et al., 2018). Reducing the use of chemical fertilizers and pesticides in agriculture is slowly becoming a reality (Olanrewaju et al., 2017). However, after BCAs are applied in the field, they cannot successfully occupy favorable competition spaces and achieve effective colonization and survival in the rhizosphere native microbial community, which is one of the main bottlenecks limiting their function (Rilling et al., 2019). In recent years, evidence has suggested that the colonization of plant roots by BCAs and the formation of root-related biofilms are key to their use as a method of biocontrol (Kolter and Greenberg, 2006; Compant et al., 2010; Chen et al., 2012; Santoyo et al., 2021).

As an important member of the BCAs family, *Bacillus* has attracted the attention of researchers (Brannen and Kenney, 1997; Ngugi et al., 2005). How this bacterium uses its advantages to colonize the roots and establish beneficial interactions with the roots of the plants is not clearly understood. In recent years, it has been found that some microorganisms with the ability to produce strong extracellular polymeric substances (EPSs) can form a biofilm adhesion structure, which makes it easier to occupy a favorable space in the competition for rhizosphere colonization space (Jayathilake et al., 2017; Karygianni et al., 2020). EPSs are mainly composed of extracellular polysaccharides, nucleic acids (eDNA and eRNA), proteins, lipids, and other biomolecules (Flemming et al., 2016). Because of the physical and chemical properties of EPSs, such as stability, viscosity, gelation, suspension ability, chelation, film formation, and water-holding capacity, biofilms can bind a large number of cells together. In this way, information exchange and interactions can occur to form a microenvironment that cooperates and co-exists with plants (Costa et al., 2018). Studies have shown that the knockout of genes related to EPSs synthesis often leads to the loss of EPS secretion ability of the strain, which makes it unable to form a biofilm structure and eventually leads to the failure of its colonization in the rhizosphere or plant tissue (Wang et al., 2008; Meneses et al., 2011).

Many reports have found that in some *Bacillus* spp. strains with the ability of EPSs secretion, γ-polyglutamic acid (γ-PGA) is the main component of its EPS (Yiyang et al., 2016; Maruzani et al., 2019). The γ-PGA biosynthesis genes are highly conserved in various *Bacillus* species. In *B. subtilis*, the biosynthesis of γ-PGA relies on the conserved operon *pgsB*-*pgsC*-*pgsA*-*pgsE* (Ashiuchi and Misono, 2002). In recent years, research on γ-PGA in agriculture has mainly focused on its biological functions, such as water retention, chelation of heavy metals, fertilizer synergism, antioxidant effect, stress resistance, and growth promotion (Wang et al., 2022). However, little is known about how γ-PGA affects biofilm formation and how it can control plant diseases. Although γ-PGA is similar to other biofilm matrix components, it is not clear whether γ-PGA plays a structural role in biofilm matrix assembly. One study showed that the *pgs*-deletion mutant of the *B. subtilis* model strain NCIB 3610 had no differences in the biofilm phenotype (Branda et al., 2006). Another study showed that γ-PGA had an effect on the biofilm phenotype when different medium conditions were used (Stanley and Lazazzera, 2005).

In this study, we screened a strain of *B. atrophaeus*, NX-12, from the rhizosphere of a cotton plant as a potential BCA. The biocontrol effect, biofilm-forming ability, and antagonistic activity of this strain against various plant pathogens were characterized. The main component of the EPS was identified as γ-PGA through the extraction and identification of the EPS. Based on the technology of CRISPR-Cas9n, we constructed a *pgsBCA*-deletion mutant (NX-12Δ*pgs*). We have demonstrated that a lack of γ-PGA led to a decrease in the biofilm formation and colonization ability of the strain, which, in turn, significantly reduced the biocontrol effect of the strain in cucumber *Fusarium* wilt. Our findings are consistent with those of a previous study that reported an inseparable relationship between biofilm formation and plant protection (Bais et al., 2004). We further showed that the extracellular matrix secreted by bacteria, especially γ-PGA, is an important factor that affects bacterial rhizosphere colonization and plant protection.

## Materials and methods

### Strain isolation

Samples were collected from the Juxin cotton farm in Hutubi County, Xinjiang, China. They were divided into four parts (root, stem, leaves, and rhizosphere soil). The rhizosphere soil samples were collected at a depth of 20 cm from the cotton planting fields. Bacterial isolation was performed using modified methods based on a previous study (Saravana Kumar et al., 2014). Tissues (root, stem, and leaves 0.2 g each) were sterilized with 75% (v/v) ethanol and 0.3% (m/v) mercuric chloride solution. The tissues were then thoroughly crushed in liquid nitrogen-containing glass beads to make them completely uniform, and they were then resuspended in a 0.9% (m/v) sterile NaCl solution. The rhizosphere soil sample (0.2 g) was mixed with a 0.9% (m/v) sterile NaCl solution, followed by shaking and mixing well for 30 min. The mixture was diluted 10 folds and plated onto Luria-Bertani (LB) agar plates. After incubation at 30°C for 24 h, colonies were picked and further purified by repeated streaking onto LB medium. The pure isolates were preserved in sterile glycerol (20% v/v) at −80°C. Subsequently, the antifungal activity was tested on PDA plates. Briefly, a 6-mm-diameter plug containing mycelium was plated at the center of the PDA plates, and 5 μl of the bacterial suspension (OD_600_ = 1.0) was patched 3 cm away from the fungus. After incubation at 28°C for 72 h, the strain with the largest antibacterial circle was selected and named NX-12 for use in further experiments.

### Characteristics of the strain NX-12

The characteristics of strain NX-12 were determined according to Bergey’s Manual of Systematic Bacteriology. The NX-12 genome was extracted using a genomic DNA purification kit and used for a polymerase chain reaction (PCR) amplification of the 16S ribosomal DNA (rDNA) gene using the primers *27F* (5′-AGAGTTTGATCMTGGCTCAG-3′) and *1492R* (5′-TACGGYTACCTTGTTACGACTT-3′). The sequences were compared to the reference sequences of other bacterial isolates deposited in the NCBI nucleotide database using the BlastN algorithm.

### Plant growth conditions

Cucumber seeds (“Jinchun No. 4”) were purchased from the Tianjin Kerun Cucumber Research Institute and surface-sterilized (Li et al., 2015). The seeds were surface-sterilized for 10 min in 30% sodium hypochlorite and rinsed three times with distilled water. The seeds were then incubated at 28°C for 48 h on a sterile wet filter paper. The sprouted seeds were then planted in sterilized soil (Laoshan National Forest Park, Nanjing, China). The initial moisture content of the soil was 30%. After 7 days of cultivation, the uniform cucumber seedlings were randomly divided into three groups: group CK, uninoculated group; group FOC, inoculated with spores of *Fusarium. oxysporum* f. sp. *cucumber* (FOC) up to a final concentration of 1 × 10^5^ CFU/g soil; and group NX-12 + FOC, which was inoculated with strain NX-12 up to a final concentration of 1 × 10^7^ CFU/g soil and spores of FOC up to a final concentration of 1 × 10^5^ CFU/g soil. The plants were then cultured for 4 weeks. The samples were then harvested. All cucumber seedlings were cultivated in a growth chamber with a 16-h photoperiod (26°C, 4,000 lx) at 65% relative humidity and fertilized twice a week with 1/4 MS liquid fertilizer.

### Plant growth measurements and physiological indices

Four weeks after inoculation with FOC, the disease severity was assessed for each plant on a 0–4 rating scale according to the percentage of defoliation (0 = healthy plant, 1 = 1–33%, 2 = 34–66%, 3 = 67–97%, and 4 = dead plant; Huang et al., 2006), and was calculated according to the following formulas: leaf wilt index (LWI) = Σ(disease score × the number of plants with that score)/(the total number of plants investigated × 4; Li et al., 2012), and biocontrol efficacy = [(LWI of control plants–LWI of treated plants)/disease incidence of control] × 100% (Chen et al., 2019). For each treatment, there were three replicates with six seedlings each. The height of cucumber plants was measured using a measuring tape. The leaf area of fully expanded leaves was recorded before flowering using a ScanMaker (i800 plus, Microtek, China). Stomatal conductance, net photosynthetic rate, transpiration rate, and intercellular carbon dioxide concentration were measured using a portable photosynthetic system (LI-800, LI-COR, United States). The MDA and POD indices were elucidated using previously described methods (Bilal et al., 2018). The quantity of FOC in the cucumber rhizosphere soil was determined as described previously (Faheem et al., 2014).

### Identification of extracellular polymers

Strain NX-12 was incubated in an LB medium at 37°C and 200 rpm for 8 h. It was then transferred into the selected medium, which contained (per liter) glucose, 30 g, sodium glutamate 30 g, (NH_4_)_2_SO_4_ 5 g, K_2_HPO_4_ 2 g, MgSO_4_ 0.1 g, and MnSO_4_ 0.03 g at an inoculum volume of 5% (v/v). The fermentation was carried out at 30°C and 200 rpm for 48 h. The EPS was purified using a previously described method (Qiu et al., 2017). The total carbohydrate content was determined by the phenol–sulfuric acid method. Simultaneously, the prepared extracellular polymer was scanned at wavelengths between 200 and 600 nm, and γ-PGA was used as a control. The amino acid composition was determined according to the procedure recommended by Amino Acid Analysis (AAA; AdvanceBio, Agilent, United States).

### Construction of the γ-PGA-deficient strain NX-12Δ*pgs* and the complementary strain NX-12Δ*pgs* (pMA5-*pgs*)

The strains and plasmids used in the present study are listed in Supplementary Table S1. The construction of mutants was based on the CRISPR-Cas9n system, as described previously (Qiu et al., 2020), and this procedure was modified appropriately. The strain was cultured overnight in a fresh NA medium at 37°C and 200 rpm, and it was then transferred into an SPI medium until the OD_600_ reached 1.5. The solution was then transferred into an SPII medium at 37°C and 100 rpm. When the OD_600_ reached 0.6, EGTA solution was added at a final concentration of 0.1 mM and incubated for 10 min. The culture broth (50 ml) was centrifuged at 6,000 × *g* for 15 min at 4°C. The cells were washed twice with ice-cold deionized water (15 ml) and then washed twice again with an equal volume of cold 10% glycerol (v/v). Finally, the cells were suspended in 10% sterile glycerol (v/v) so that the cell density was 1 × 10^10^ CFU/ml. Precooled plasmid DNA (100 ng) and competent cells (100 μl) were mixed gently and then transferred into pre-cooled 2-mm electroporation cuvettes. The sample was then exposed to a single electrical pulse (2 kV, 4 ms). Next, an NA medium (800 μl) was immediately added. After growth at 30°C and 200 rpm for 3 h, the cells were plated onto LB agar (Spec, 25 μg/ml; Kar, 50 μg/ml; Cm, 10 μg/ml if necessary). The PCR primers listed in Supplementary Table S2 were used to screen and identify positive colonies. Specifically, overnight cultured cells were transferred to a new resistant LB medium containing 1% of the inoculum. When the OD_600_ reached 0.8, IPTG was added at a final concentration of 1 mM. After induction at 30°C and 200 rpm for 10 h, the positive clones were grown at 30°C for 12 h in LB medium containing spectinomycin and then subcultured for more than 20 generations. A single colony with a dry morphology was selected for culture. The results were verified by PCR and synchronized with the sequencing results.

The *pgsBCA* gene was amplified using the primers pMA5-*pgsBCA*-F and pMA5-*pgsBCA*-R from the NX-12 genome, and a fragment of the plasmid pMA5 was used to construct the recombinant plasmid pMA5-*pgsBCA* by In-Fusion cloning. The mutant NX-12Δ*pgs* was complemented with pMA5-*pgsBCA*. Analysis of the EPS from NX-12Δ*pgs* (pMA5-*pgs*) was performed as described above.

### Comparison of the antibacterial activity and biocontrol abilities of NX-12 and NX-12Δ*pgs*

The antibacterial activity of the wild isolate toward five important types of *Fusarium oxysporum* (FO; f. sp. *cucumerium, strawberry, cotton, lotus root,* and *watermelon*) was measured. Strains were cultured in an LB medium for 12 h at 30°C and 200 rpm. The culture broth’s pellets were then washed with a PBS buffer (pH 7.0) and resuspended to OD_600_ = 1.0. A suspension of the wild isolate of NX-12 was placed around the fungal inocula at a distance of 3 cm. After incubation at 28°C for 72 h, the zones of inhibition were measured, as described previously (Berg et al., 2005). The fungus FOC with the best inhibition effect was used for the comparison of the antibacterial activities between NX-12 and NX-12Δ*pgs*, and the experimental steps were consistent with those described above.

The plants were grown under the same conditions as those described in Plant growth conditions, with some modifications. Uniform seedlings were randomly divided into four groups. Group CK was the uninoculated group; group FOC was inoculated with spores of the FOC up to a final concentration of 1 × 10^5^ CFU/g soil; group NX-12 + FOC was inoculated with strain NX-12 up to a final concentration of 1 × 10^7^ CFU/g soil; and group NX-12Δ*pgs* + FOC was inoculated with strain NX-12Δ*pgs* up to a final concentration of 1 × 10^7^ CFU/g soil. After 4 weeks of cultivation, the samples were collected, and the LWI, dry weight, and height were measured.

### qPCR analysis

The prediction and analysis of the secondary metabolite genes of the strain were analyzed using antiSMASH (https://antismash.secondarymetabolites.org/). The complete genome sequence of NX-12 was determined using Novogene (Beijing, China). According to the standard protocol, bacterial RNA was extracted using an RNA isolation kit (RC112-01, Vazyme). It was necessary to lyse the cell wall with lysozyme at a final concentration of 20 mg /mL before extraction. The primer sequences used for qPCR are listed in Supplementary Table S2. qPCR was performed according to the recommended protocol (R323-01/Q711-02/Q711-03, Vazyme). The 2^-ΔΔCT^ method was used to analyze the qPCR data (Kenneth and Thomas, 2002).

### Biofilm formation assay

Biofilm formation was determined by crystal violet staining (Hsueh et al., 2006) with some modifications. NX-12 and NX-12Δ*pgs* were grown in LB broth at 30°C and 200 rpm overnight to generate inoculum cultures. They were then adjusted to an optical density at 600 nm (OD_600_) of 0.01. Specifically, the modified Msgg medium (0.005 M potassium phosphate buffer, 0.1 M Mops, 0.002 M MgCl_2_, 0.7 M CaCl_2_, 0.05 M MnCl_2_, 0.05 M FeCl_3_, 0.002 M VB_1_, 1.2% (m/v) glucose, and 1.2% (m/v) sodium glutamate) was pre-formulated. Next, 2 ml of modified Msgg medium was added to the wells of polystyrene 24-well plates, followed by incubation at 30°C for 24 h. The planktonic bacteria were removed and the wells were washed with distilled water and air-dried. The remaining biofilm cells were stained with 2 ml of 0.3% crystal violet for 10 min, then washed with distilled water, and were finally air-dried. The crystal violet in the biofilm cells was solubilized with 2 ml of 70% ethanol, and the optical density at 570 nm (OD_570_) was measured.

### Rhizosphere colonization assay

The general methods for the rhizosphere colonization assay followed published protocols (Tian et al., 2021) with some modifications. NX-12-gfp, NX-12Δ*pgs*-gfp, and NX-12Δ*pgs*-gfp (PMA5-*pgsBCA*) were constructed according to the protocol described in Construction of the γ-PGA deficient strain NX-12Δ*pgs* and the complementary strain NX-12Δ*pgs* (pMA5-*pgs*). The uniform seedlings were divided into four groups: NX-12, NX-12Δ*pgs*, NX-12Δ*pgs* + γ-PGA, and NX-12Δ*pgs* (PMA5-*pgsBCA*). The cucumber seeds were cultured in plastic pots (70 mm × 70 mm × 75 mm) containing 15 g sterilized vermiculite, and the pots were incubated in moist chambers (26°C/23°C day/night temperatures, 4,000 Lux light for 16 h/day, and 65% relative humidity). The pots were watered weekly with 1/4 MS. After 15 days of incubation, 5 ml of cell suspension containing freshly cultivated *B. atrophaeus* cells (1.0 × 10^8^ CFU/ml) supplemented with γ-PGA (0.1 mg/ml) was added to the pot by pouring onto the surrounding root. After another 3 days of incubation, the roots of the seedlings were removed and rinsed with sterilized water. A 0.2-g root ripening zone for each sample was collected and immediately stored in a sterile Eppendorf tube for fluorescence microscopy (FM) and plate recovery counting.

The green fluorescent protein (GFP) gene was biosynthesized using GenScript (Nanjing, China) and amplified using the primer pairs pM-GFP-F and pM-GFP-R. pMA5-GFP was constructed using In-Fusion cloning as described above. Next, pMA5-GFP was electroporated into NX-12 and NX-12Δ*pgs*. The cucumber seedlings were treated as described above, and the roots were observed under a fluorescence microscope to detect the presence of the bacteria.

## Results

### Isolation and identification of *Bacillus atrophaeus* NX-12

The initial purpose of this work was to screen candidate strains for BCAs. We isolated strains from both four parts (root, stem, leaves, and rhizosphere soil) of samples and the strains co-existing in the four parts were selected as the target strains. Supplementary Figure S1 shows the microbiological of the strain NX-12. This strain is a Gram-positive, motile, spore-forming, rod-like bacterium. A partial 16S rRNA gene sequence analysis (1,416 bp) demonstrated that strain NX-12 was most likely *B. atrophaeus* strain JCM9070 (98%), indicating that strain NX-12 belongs to the species *B. atrophaeus* (Supplementary Figure S1). Therefore, we designated this strain as *B. atrophaeus* NX-12.

### Strain NX-12 exhibits high biocontrol efficacy against *Fusarium* wilt on cucumber

**Figure 1:**
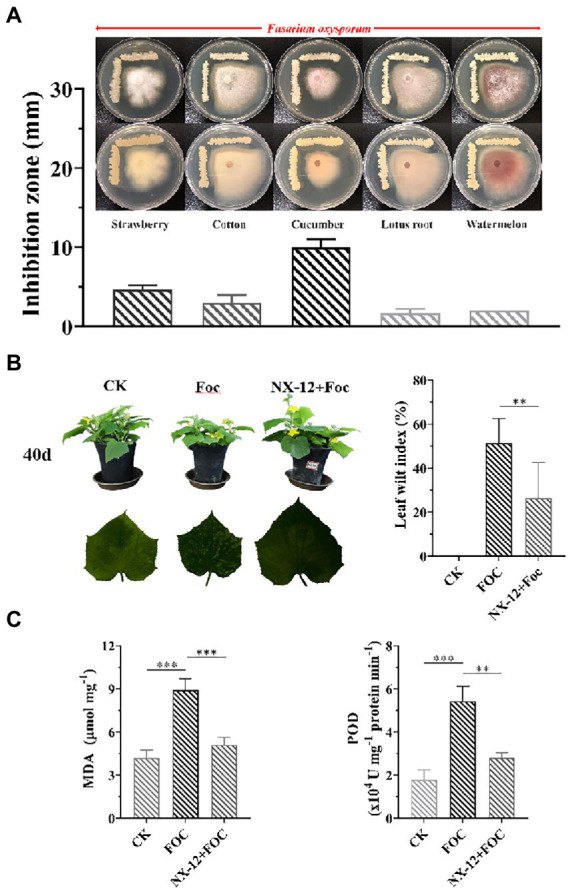
Antifungal activity and biocontrol efficacy of *Bacillus atrophaeus* NX-12. **(A)** The inhibitory effect of NX-12 on *Fusarium oxysporum* of different plant specialization types; **(B)** Biocontrol efficacy of strain NX-12 against *Fusarium* wilt in cucumber; **(C)** Activities of MDA and POD in Cucumber Leaves. Error bars represent standard deviations. ^**^ indicated value of *p* < 0.01; ^***^ indicated value of *p* < 0.001.

To evaluate the inhibition ability of the strain to the pathogen of *Fusarium* wilt, five *Fusarium* wilt Pathogens from different plant specialization types were used to test, in which NX-12 had the strongest effect on FOC (Figure 1A). The biocontrol efficacy of *Fusarium* wilt on cucumber seedlings was also verified. *Fusarium* wilt in cucumber plants peaks 4 weeks after the challenging inoculation with FOC. The stomatal conductance, net photosynthetic rate, and transpiration rate of cucumber seedlings in the pathogen treatment group were the lowest among the three treatments, which were decreased by 65.05, 41.73, and 64.74%, respectively, when compared with the control group. At the same time, the intercellular carbon dioxide concentration was 21.91% lower than that in the control group. However, the above conditions were significantly changed by the treatment with NX-12 + FOC, which meant that the above values changed to be 56.75, 103.14, and 55.12% of the control group, respectively (Supplementary Figure S1). The leaf wilt index (LWI) value obtained in the seedlings treated with FOC was 51.39%, which was significantly higher than the LWI of the NX-12 + FOC group (26.39%; Figure 1B). The contents of MDA (8.91 μmol mg^−1^ for FOC versus 5.11 μmol mg^−1^ for NX-12 + FOC) and POD (5.42 U mg^−1^ protein min^−1^ for FOC versus 2.81 U mg^−1^ protein min^−1^ for NX-12 + FOC) indicated that strain NX-12 exhibits great biocontrol efficacy against *Fusarium* wilt in terms of the physiological indicators (Figure 1C).

### Main component of the extracellular polymeric substances is γ-PGA

**Figure 2:**
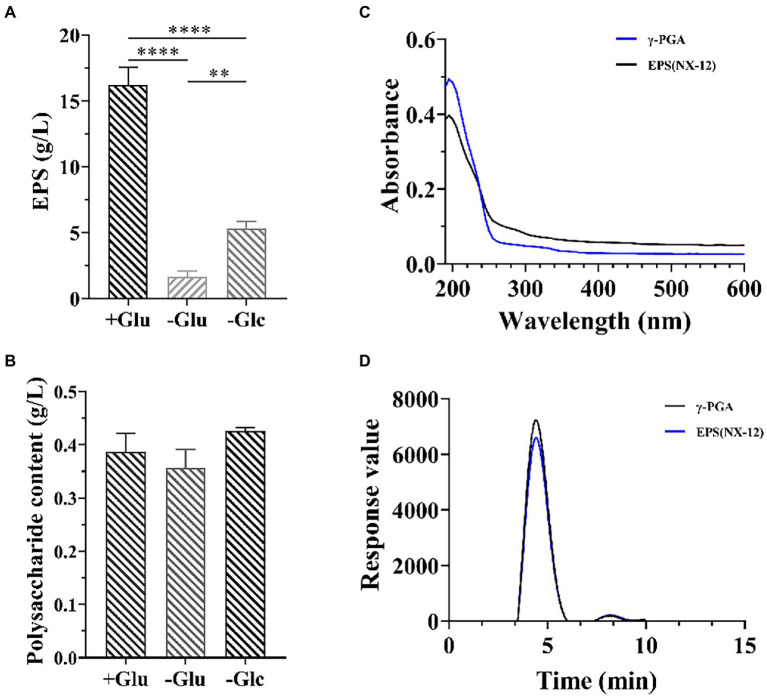
Isolation and Identification of EPS from NX-12. **(A)** Comparison of the production of EPSs by fermentation; **(B)** Comparison of the production of extracellular polysaccharide by fermentation; **(C)** Spectral analysis of EPS from NX-12 and γ-PGA. **(D)** Comparison of monomer liquid phase analysis after hydrolysis of EPS and γ-PGA; Error bars represent standard deviations. ^**^ indicated value of *p* < 0.01; ^****^ indicated value of *p* < 0.0001.

Because strain NX-12 is a robust biofilm-forming strain, it can form a strong biofilm on the surface of solid and liquid (Supplementary Figure S2). The formation of biofilm is closely related to the secretion of extracellular matrix. We then measured the yield of its EPSs, the yield of which reached 16.8 g/l in the selected medium. At the same time, it was also proved that the effect of nitrogen source on yield was greater than that of carbon source (Figure 2A). However, there was no significant difference in the polysaccharide content of the EPS, regardless of the medium (Figure 2B). This also prompted us to think about polypeptides rather than polysaccharides. Spectral analysis revealed that EPS and γ-PGA had a consistent maximum absorption peak at 209 nm (Figure 2C). Subsequently, the results of High-Performance Liquid Chromatography (HPLC) analysis verified that the content of glutamate after hydrolysis reached 89.5% of the EPSs (Figure 2D).

### Knockout of *pgsBCA* significantly reduced the yield of the EPS

**Figure 3:**
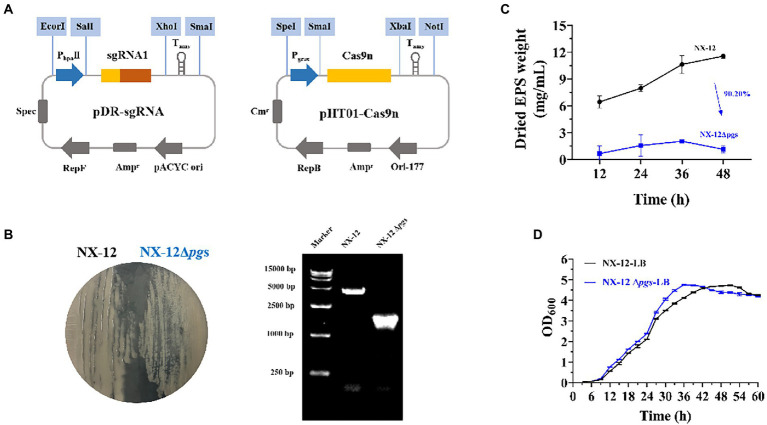
Knockout of *pgsBCA* based on CRISPR-Cas9n system. **(A)** Construction of double plasmid system; **(B)** Comparison of colony morphology and verification by PCR after knockout; **(C)** Yield of EPS between NX-12 and NX-12Δ*pgs*; **(D)** Growth curves of NX-12 and NX-12Δ*pgs* in LB medium.

To better understand the function of γ-PGA, the γ-PGA synthase gene *pgsBCA* of NX-12 was knocked out using the Crispr-Cas9n system (Figure 3A). It can be clearly seen that the colony morphology on the plate changed from wet to dry. PCR screening was used to verify whether the target gene fragment (*pgsBCA*) had been effectively deleted. A 2048-bp PCR product was amplified using the mutant chromosome DNA as a template, which was 2,813 bp less than the PCR product using the NX-12 chromosome DNA as a template (Figure 3B). The mutant strain was designated *B. atrophaeus* NX-12Δ*pgs*. The EPS yield of NX-12Δ*pgs* was 90.2% lower than that of NX-12 (Figure 3C). There was no significant difference between the wild-type and mutant bacteria in the LB medium (Figure 3D).

### The transcription levels of genes related to antimicrobial peptide synthesis and glutamate metabolism were different between wild and the mutant strain

**Figure 4:**
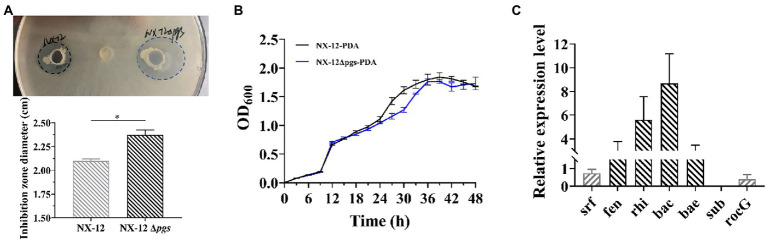
Comparison of the transcription levels of genes related to antimicrobial peptide synthesis between NX-12 and NX-12Δ*pgs*. **(A)** Comparison of antibacterial ability of NX-12 and NX-12Δ*pgs* against FOC *in vitro*; **(B)** Growth curves of NX-12 and NX-12Δ*pgs* in PDB medium; **(C)** Transcription levels of synthetic genes related to antimicrobial peptides in NX-12 and NX-12Δ*pgs*. Error bars represent standard deviations. ^*^ indicated value of *p* < 0.05.

In order to confirm whether the knockout of *pgsBCA* has an effect on the antifungal ability of the strain, we verified the antifungal ability of wild bacteria and mutant bacteria by punching holes on the plate. Contrary to our expectations, it can be clearly seen that NX-12Δ*pgs* had significantly improved inhibition activity against pathogenic fungi when compared with NX-12 (Figure 4A; Supplementary Figure S3). However, the growth curve of NX-12 and NX-12Δ*pgs* in the PDA liquid medium showed no significant difference within 48 h (Figure 4B). We therefore tested the transcription of genes related to antimicrobial peptide synthesis as well as glutamate transferase. Six lipopeptide antibiotics were predicted according to the antiSMASH website, and key synthetic genes were identified using the NCBI comparison database. The results of qPCR showed that when compared with NX-12, the synthesis of fengycin, rhizocticin A, bacillibactin, and bacillaene genes of NX-12Δ*pgs* was significantly upregulated. No significant differences were observed between surfactin and subtilisin A. The transcription level of the glutamate dehydrogenase gene *rocG*, which is related to nitrogen metabolism (Feng et al., 2015), was significantly decreased (Figure 4C). These results suggest the enhancement of antifungal ability *in vitro*.

### The biocontrol ability of NX-12Δ*pgs* against Cucumber *Fusarium* wilt decreased significantly

**Figure 5:**
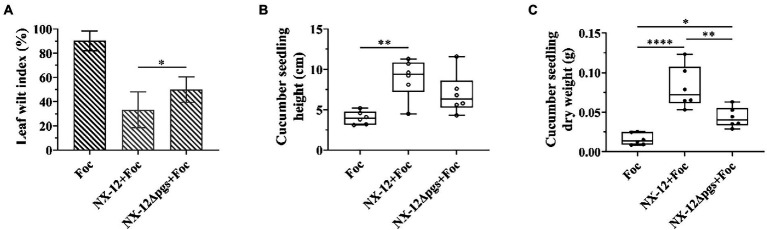
The biological control ability of NX-12Δ*pgs* decreased significantly. **(A)** The leaf wilt index (LWI) under different treatments; **(B)** Seeding height of cucumber treated by NX-12 and NX-12Δ*pgs* in controlling Fusarium wilt; **(C)** Seeding dry weight of cucumber treated by NX-12 and NX-12Δ*pgs* in controlling Fusarium wilt. Error bars represent standard deviations. ^*^ indicated value of *p* < 0.05; ^**^ indicates value of *p* < 0.01; ^****^ indicates value of *p* < 0.0001.

As described above, the deletion of *pgsBCA* led to the enhancement of antifungal activities. Therefore, we wondered whether wild bacteria and mutant bacteria have the same effect in plant biocontrol as *in vitro*. The biocontrol abilities of NX-12 and NX-12Δ*pgs* in cucumber seedlings were tested. Contrary to the antibacterial effect *in vitro*, the leaf wilt index (LWI) value obtained in the seedlings treated with strain NX-12Δ*pgs* was 50%, which was significantly higher than the LWI of the NX-12 group (33.3%). Whether the seedlings were treated with NX-12 or NX-12Δ*pgs*, the LWI of cucumbers after treatment was significantly lower than that of the FOC group (90.3%; Figure 5A). In addition, the height and dry weight of the cucumber seedlings were measured. Compared to the NX-12Δ*pgs* + FOC group, both the height and dry weight were significantly increased in the NX-12 + FOC group (Figures 5B,C). This indicated that strain NX-12 was more effective than strain NX-12Δ*pgs* at suppressing cucumber *Fusarium* wilt.

### Synergistic action between γ-PGA and NX-12 to enhance the ability of colonization and biocontrol

**Figure 6:**
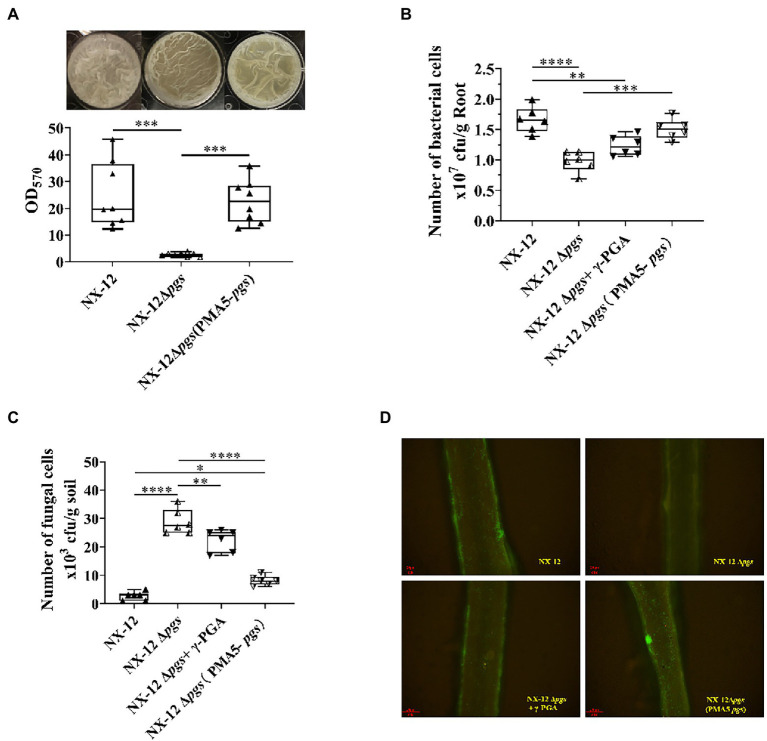
γ-PGA helps colonize and control pathogens. **(A)** Difference in biofilm formation between NX-12 and NX-12Δ*pgs in vitro*; **(B)** Number of bacteria cells colonized on the root surface of cucumber under the treatments of NX-12, NX-12Δ*pgs*, NX-12Δ*pgs* + γ-PGA, and NX-12Δ*pgs* (pMA5-*pgs*); **(C)** Number of fungal cells colonized on the root surface of cucumber under the treatments of NX-12, NX-12Δ*pgs*, NX-12Δ*pgs* + γ-PGA, and NX-12Δ*pgs* (pMA5-*pgs*); **(D)** Colonization of fluorescent strains in the rhizosphere. Error bars represent standard deviations. ^*^ indicates value of *p* < 0.05; ^**^ indicates value of *p* < 0.01; ^***^ indicated value of *p* < 0.001; ^****^ indicates value of *p* < 0.0001.

Since the function of BCA is related to the interaction between host plants, we further hypothesized that NX-12 could realize the function of biological control by enhancing the formation of biofilm and promoting its colonization in the rhizosphere with the help of secreted γ-PGA. The biofilm-forming ability of NX-12 was significantly higher than that of NX-12Δ*pgs* (Figure 6A). NX-12 could form a clear thick layer of biofilm (OD_570_ = 20.44), whereas the biofilm formation ability of NX-12Δ*pgs* (OD_570_ = 2.57) was significantly diminished, which was only 12.6% when compared to NX-12. Under pot soil conditions, colonization of NX-12 and NX-12Δ*pgs* in the rhizosphere was determined by plate counting and fluorescence observation. It was found that NX-12Δ*pgs* (0.98 × 10^7^ CFU/g root) failed to colonize roots as effectively when compared to NX-12 (1.66 × 10^7^ CFU/g root). To further support the idea that γ-PGA plays a key role in rhizosphere colonization, the mutant NX-12Δ*pgs* was complemented with pMA5-*pgsBCA* and we named NX-12Δ*pgs* (pMA5-*pgs*). The EPS yield of NX-12Δ*pgs* (pMA5-*pgs*; 11.17 mg/ml) recovered significantly compared to that of NX-12Δ*pgs* (2.77 mg/ml; Supplementary Figure S4). As expected, NX-12Δ*pgs* (pMA5-*pgs*) restored most of its biofilm formation (Figure 6A) and colonization ability. At the same time, the exogenous addition of γ-PGA improved the colonization ability of NX-12Δ*pgs* (1.23 × 10^7^ CFU/g root), although it did not reach the level of NX-12Δ*pgs* (pMA5-*pgs*; 1.51 × 10^7^ CFU/g root; Figure 6B). The FOC population was monitored using plate counting and remained high (28.83 × 10^3^ CFU/g soil) in the NX-12Δ*pgs* group compared to that in the NX-12 group (2.67 × 10^3^ CFU/g soil). However, the FOC population in the rhizosphere was significantly reduced by the application of NX-12Δ*pgs* (pMA5-*pgs*; 7.8 × 10^3^ CFU/g soil). Exogenous addition of γ-PGA also slightly reduced the FOC population to 22.33 × 10^3^ CFU/g soil (Figure 6C).

Fluorescence microscopy images also confirmed that γ-PGA significantly affected the colonization ability of NX-12 (Figure 6D).

## Discussion

Rhizosphere bacteria play a key role in protecting plants and promoting plant growth and health (Berendsen et al., 2012). *Bacillus subtilis*, as well as other *Bacilli*, have been used as important BCAs in agriculture (Nagórska et al., 2007; Ongena and Jacques, 2008; Liu et al., 2021). The newly isolated strain, *B. atrophaeus* NX-12, demonstrated strong antifungal efficacy toward FOC *in vitro* and biocontrol activities *in situ*. The mechanism by which *B. atrophaeus* exerts a strong biocontrol activity in the rhizosphere is not well understood. Previous studies have provided evidence that the production of antimicrobial agents, biofilm formation, and triggering host systemic resistance can contribute to the biocontrol activities of *Bacillus* (Bais et al., 2004; Chen et al., 2019). Here, we have focused on the role of biofilm formation in plant biocontrol, and we have provided several types of evidence documenting its importance.

In view of the high yield of γ-PGA in NX-12, which is not common in most *Bacillus* sp. strains, we first knocked out the key genes for γ-PGA synthesis, *pgsBCA*, through CRISPR-Cas9n. A strain NX-12Δ*pgs* with low γ-PGA yield was obtained and used to study the differences between NX-12Δ*pgs* and wild strains. Interestingly, NX-12Δ*pgs* showed better antifungal activity against the pathogen than NX-12, which was not as expected. Previous studies have found that *Bacillus* species have become the most successfully commercialized biocontrol agents owing to their ability to produce a broad spectrum of antimicrobial secondary metabolites (Koumoutsi et al., 2004; Sansinenea and Ortiz, 2011; Xingshan et al., 2021). These substances can inhibit many phytopathogens, including fungi and bacteria (Bais et al., 2004; Koumoutsi et al., 2004; Sansinenea and Ortiz, 2011; Liu et al., 2021). We predicted some secondary metabolites that NX-12 might produce according to the secondary metabolite prediction analysis website antiSMASH and mined its key genes using NCBI. qPCR analysis showed that genes related to antimicrobial peptide synthesis (*fen, rhi, bac, and bae*) were significantly upregulated in the mutants, while *rocG*, a gene related to glutamate synthesis (Feng et al., 2015), was significantly downregulated. Therefore, we hypothesized that the increased antifungal ability of mutant bacteria was due to the fact that more nitrogen was used for the synthesis of antimicrobial substances, while in wild bacteria, more nitrogen was used for the synthesis of γ-PGA.

On the contrary, the biocontrol effect of NX-12Δ*pgs* was significantly decreased *in situ*. This showed that antifungal ability was not the only criterion for considering biocontrol ability. Many studies have shown that the effective colonization of microorganisms in plant rhizosphere determines whether they can play the corresponding biological functions. Biofilm formation is critical for bacterial rhizosphere colonization (Cao et al., 2011; Allard-Massicotte et al., 2016). Here, we found that the biofilm-forming capacity of NX-12Δ*pgs* was significantly lower than that of NX-12. Although extracellular polysaccharides are thought to be the main component of biofilms; in fact, in some strains with high yield of γ-PGA, it is the main component of biofilms (Stanley and Lazazzera, 2005). In addition, we found that the number of rhizosphere colonization of NX-12 was significantly higher than that of NX-12Δ*pgs* due to its strong biofilm-forming ability. Interestingly, we also found that FOC colonization in rhizosphere in NX-12 group was significantly less than that in NX-12Δ*pgs* group. These results indicated that γ-PGA enhanced the biofilms formation of NX-12, promoted the colonization of NX-12 in the rhizosphere, and occupied the dominant ecological niche, thus enhancing the resistance of plants to biological stress through this indirect way.

In conclusion, our research shows that the newly isolated *B. atrophaeus* NX-12 with high yield of γ-PGA owns strong antifungal ability against FOC *in vitro* and biocontrol effects *in situ*. It is found that γ-PGA enhances plant tolerance to biotic stress by promoting the formation of NX-12 biofilm and rhizosphere colonization ability. Our results reveal that the effective colonization of BCAs in the rhizosphere is very important for its function. Our work broadens the research direction of biofilms and has enhanced our knowledge of the application of γ-PGA in biocontrol.

## Data availability statement

Strain Bacillus atrophaeus NX-12 presented in the study is deposited in the CGMCC repository, accession number 22125.

## Author contributions

JX, YG, LS, HX, and PL conceived and designed study. JX and YQ contributed to the new methods or models. JX and TT performed the experiments. JX performed the data collection and analysis and the first draft of the manuscript. RW, YG, LS, HX, and PL assisted JX in revising the previous versions of the manuscript. All authors contributed to the article and approved the submitted version.

## Funding

The research was funded by the National Key Research and Development Program of China (2021YFC2101700), the National Natural Science Foundation of China (42177271), the Jiangsu Agricultural Science and Technology Innovation Fund [CX (21) 3158], the Key Research and Development Project of Jiangsu Province (BE2019390), and the Jiangsu Synergetic Innovation Center for Advanced Bio-Manufacture (XTB2202).

## Conflict of interest

The authors declare that the research was conducted in the absence of any commercial or financial relationships that could be construed as a potential conflict of interest.

## Publisher’s note

All claims expressed in this article are solely those of the authors and do not necessarily represent those of their affiliated organizations, or those of the publisher, the editors and the reviewers. Any product that may be evaluated in this article, or claim that may be made by its manufacturer, is not guaranteed or endorsed by the publisher.

## Supplementary material

The Supplementary materials for this article can be found online at: https://www.frontiersin.org/articles/10.3389/fmicb.2022.972393/full#supplementary-material
