# Supplementary material for: Secretion of poly-γ-glutamic acid by Bacillus atrophaeus NX-12 enhanced its root colonization and biocontrol activity
Source: Front Microbiol. 2022 Jul 29;13:972393. doi: 10.3389/fmicb.2022.972393 (PMC9372288; doi:10.3389/fmicb.2022.972393)
Supplement: Supplementary file 1 [file Data_Sheet_1.docx]

**Supplementary Materials**

**Secretion of poly-γ-glutamic acid by *Bacillus atrophaeus* NX-12 enhanced its root colonization and biocontrol activity**

Jian Xue^1^, Tong Tong^1^, Rui Wang^1^, Yibin Qiu^1^, Yian Gu^1^, Liang Sun^1,*^, Hong Xu^1,*^, Peng Lei^1,*^

^1^ State Key Laboratory of Materials-Oriented Chemical Engineering, College of Food Science and Light Industry, Nanjing Tech University, Nanjing 211816, China

***Corresponding author**

Liang Sun; Nanjing Tech University; Tel/Fax: +86-25-58139433; E-mail address: sunl@njtech.edu.cn;

Hong Xu; Nanjing Tech University; Tel/Fax: +86-25-58139433; E-mail address: [xuh@njtech.edu.cn;](mailto:xuh@njtech.edu.cn;)

Peng Lei; Nanjing Tech University; Tel/Fax: +86-25-58139433; E-mail address: lei-peng@njtech.edu.cn;

**Supplementary Tables**

**Table S1 Strains and Plasmids Used in this Study**

| **Strains or plasmids** | **Relevant properties** | **Source** |
| --- | --- | --- |
| Strains |  |  |
| *E. coli* DH5α | F-, φ80dlavZΔM1, Δ(lacZYA-argF) U169, deoR, recA1, endA1, hsdR17(rk-, mk+), phoA, supE44, λ-thi-1, gyrA96, relA1 | this lab |
| *E. coli* GM2163 | F−, ara-14leuB6thi-1fhuA31lacY1tsx-78galK2galT22supE44hisG4rpsL136 (Strr)xyl-5mtl-1dam13: Tn9 (Camr)dcm-6mcrB1hsdR2mcrA | this lab |
| *B. atrophaeus* NX-12 | Wild type | this study |
| *B. atrophaeus* NX-12Δ*pgs* | NX-12Δ*pgs*BCA | this study |
| *Fusarium oxysporum* | Pathogen of plant Fusarium Wilt, Strawberry/ cotton/ cucumber/lotus root/watermelon specialized type | this lab |
| *Fusarium graminearum* | Pathogen of wheat Root Rot | this lab |
| *Alternaria alternata* | Pathogen of cucumber black spot | this lab |
| *Rhizoctonia solani* | Pathogen of rice sheath blight | this lab |
| Plasmids |  |  |
| pMA5 | Ampr, Kmr, *E. coli*-*B. subtilis* shuttle vector | this study |
| pHT01 | Ampr, Cmr, *E. coli*-*B. subtilis* shuttle vector | this study |
| pDR | Ampr, Spec, temperature-sensitive *E. coli*-*B. subtils* shuttle vector with the removal of cre gene | this study |

**Table S2 Oligonucleotide primers used in this study**

| **Gene/Primer** | **Forward primer (5’-…-3’)** | **Reverse primer (5’-…-3’)** |
| --- | --- | --- |
| 16S rRNA | agagtttgatcctggctcag | ggttaccttgttacgact |
| pM-*gfp* | AAAAGGAGCGATTTACATATGATGACCACCTTTAAAATCGAGAGC | GAGCTCGACTCTAGAGGATCCTTACATATCACGCGGCGCG |
| pM-*Sgrna* | aaaaggagcgatttacatatgTTCGCAGCCATGCTTATTACgttttagagctagaaatagcaagtt | GGCAAGAATTCATCTGATGAGAAACTCCTTAAAAAAGCACCGACTCGGTGCCACTTTTTC |
| pM-*Pgs*L | GAAAAAGTGGCACCGAGTCGGTGCTTTTTTAAGGAGTTTCTCATCAGATGAATTCTTGCC | CCGTCTTCGACATCCCAGACGAAATTCGAGTCGCCTTTTTTCTAATATTCCGATGACTAC |
| pM-*Pgs*R | GTAGTCATCGGAATATTAGAAAAAAGGCGACTCGAATTTCGTCTGGGATGTCGAAGACGG | gagctcgactctagaggatccAACGCCTTTTGTCTGTGAGCTGTG |
| pDR-*Sgrna* | aaaaggagcgatttagtcgacTTCGCAGCCATGCTTATTACgttttagagctagaaatagcaagtt | tgtttttttattaccctcgagAACGCCTTTTGTCTGTGAGCTGTG |
| pM-*Out* | ATCAATGATATCGCAAAGTTCATTCTTTTC | CTCTCCTGCCGTCCATTGCTGTTCTGCCGA |
| *Bae* | ATGTCGGGCGTGCGTTTT | TTGCGATTGCTCCCTGACC |
| *Fen* | ATGTGTGGACATTGTTCCATTCA | CCCCTTCTTCTTTCAGCAGCA |
| *Bac* | ATGATCCTACAGTGGCCGGTC | GGCTTGGTCCTTCCAGACAGT |
| *Sub* | ATGCCGCTGCATGCGACC | TAAAACGCCTTTTTCGTACAGCA |
| *Srf* | ATGTAAACTTACCTAAACTGACAGAAAAGC | GGACTTTTACCTGATTATCAATTCTTCC |
| *Rhi* | ATGCCGGGAGAGCTCTGTG | ATTAGGTAACCATCGGACGAGATC |
| *ROCG* | ATGTAGTAGGGCCATTTGCTGC | TATTCACAGATGTCATTTCGAATCAA |

**Supplementary Figures**


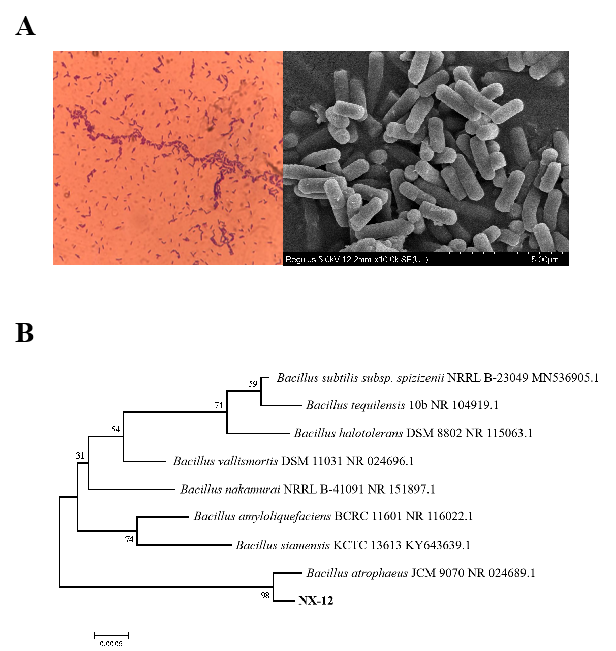


**Figure S1 Identification of strain NX-12.**

**A**: Gram staining and scanning electron microscopy of NX-12; **B**: Phylogenetic tree based on 16S rDNA gene sequence.


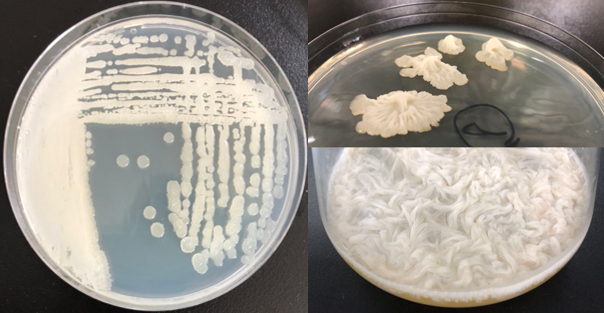


**Fig.S2 Colony morphology and biofilm formation of NX-12**


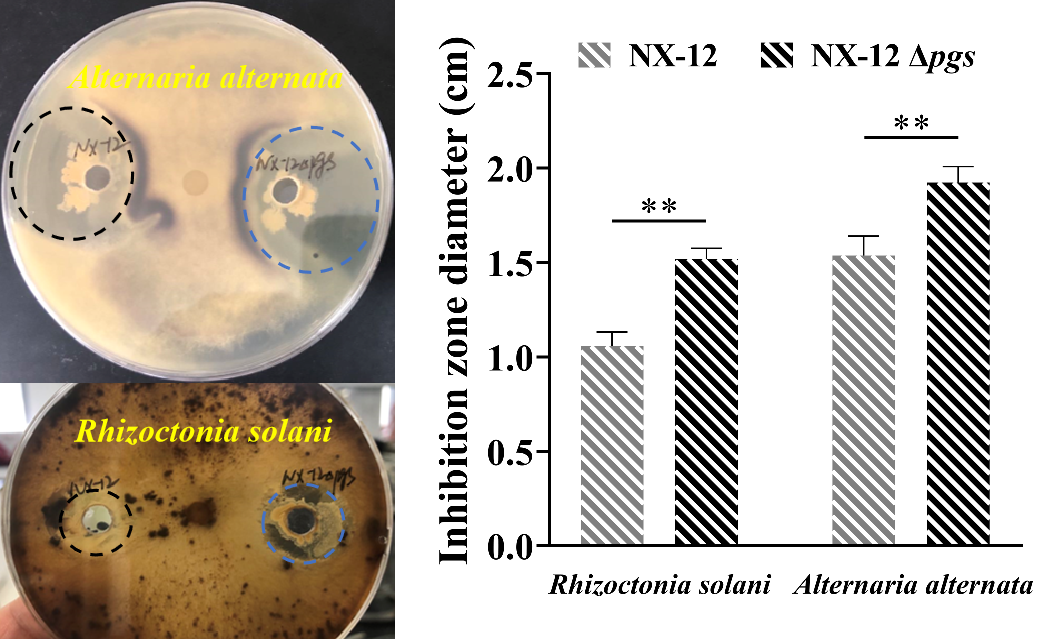


**Fig.S3 Comparing the antibacterial ability of NX-12 and NX-12Δ*pgs* on *Alternaria* *alternata* and *Rhizoctonia solani*. ** indicates p value < 0.01.**


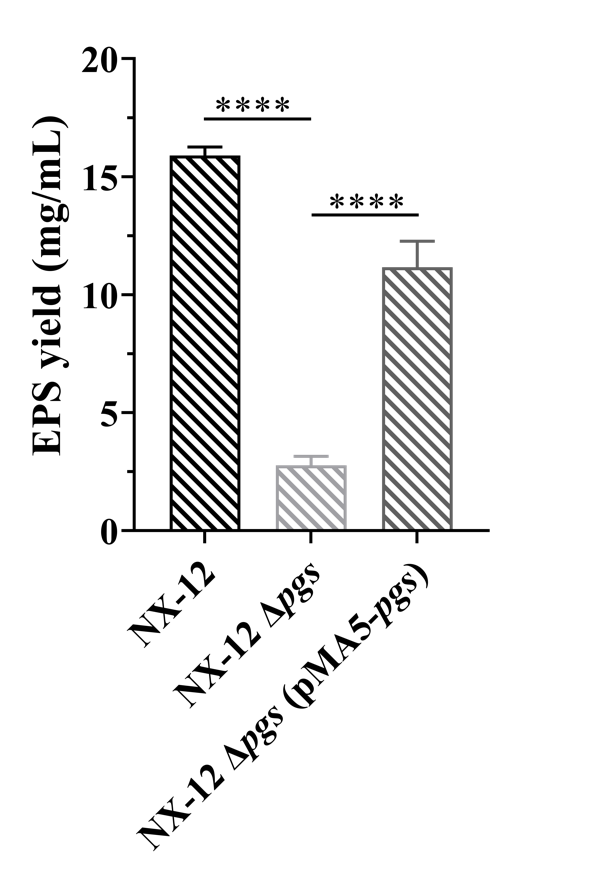


**Fig.S4 Differences in EPS production between NX-12, NX-12Δ*pgs* and *pgs*-complementary strains NX-12Δ*pgs* (pMA5-*pgs*). **** indicates p value < 0.0001.**
